# Supplementary material for: New Genes Identified as Modulating Salt Tolerance in Maize Seedlings Using the Combination of Transcriptome Analysis and BSA
Source: Plants (Basel). 2023 Mar 15;12(6):1331. doi: 10.3390/plants12061331 (PMC10053919; doi:10.3390/plants12061331)
Supplement: Supplementary file 1 [file plants-12-01331-s001.zip › plants-2226353-supplementary.pdf]

Table S1. The overlap of ED and Delta-SNP index.

Chr	Pos	delta-SNP-index	ED^4	REF	ALT	Gene_ID
**2**	3587536	0.52	0.301	TAATTCAGGTTGTGATCATTTACGCAACG	T	Zm00001d001962
**2**	168261817	0.69	0.878	G	A	Zm00001d005297
**2**	168260695	0.48	0.215	T	C	Zm00001d005297
**2**	183811241	0.51	0.275	G	A	Zm00001d005699
**2**	186199226	0.47	0.202	C	T	Zm00001d005752
**8**	9259117	0.55	0.361	G	A	Zm00001d008458
**8**	9259108	0.49	0.235	A	C	Zm00001d008458
**8**	9259098	0.46	0.186	A	G	Zm00001d008458
**8**	15013849	0.52	0.287	C	T	Zm00001d008623
**8**	15013842	0.51	0.281	C	T	Zm00001d008623
**8**	88655940	0.52	0.292	C	G	Zm00001d009903
**8**	96481004	0.5	0.247	G	A	Zm00001d010037
**8**	96480981	0.48	0.204	G	A	Zm00001d010037
**8**	145144836	0.46	0.184	C	G	Zm00001d011268
**8**	161465492	0.55	0.355	T	G	Zm00001d011779
**8**	161465489	0.5	0.241	A	T	Zm00001d011779
**8**	178737021	0.52	0.298	G	A	Zm00001d012694
**5**	181497196	0.5	0.241	G	A	Zm00001d016963
**5**	181497199	0.5	0.241	T	C	Zm00001d016963
**5**	181497213	0.49	0.24	C	T	Zm00001d016963
**5**	181497215	0.48	0.215	G	A	Zm00001d016963
**7**	9814722	0.55	0.372	C	T	Zm00001d018937
**7**	9814724	0.53	0.308	G	A	Zm00001d018937
**7**	10334086	0.46	0.184	TA	T	Zm00001d018950

**7**	26623736	0.51	0.269	C	T	Zm00001d019299
**7**	27320622	0.48	0.221	A	G	Zm00001d019314
**7**	27985487	0.5	0.248	T	C	Zm00001d019327
**7**	33480773	0.63	0.633	T	C	Zm00001d019432
**7**	33480775	0.63	0.633	G	A	Zm00001d019432
**7**	33480531	0.59	0.481	A	G	Zm00001d019432
**7**	33480518	0.59	0.47	C	T	Zm00001d019432
**7**	33480567	0.55	0.35	G	T	Zm00001d019432
**7**	35830274	0.53	0.323	C	G	Zm00001d019476
**7**	37502895	0.6	0.524	T	C	Zm00001d019495
**7**	41794218	0.51	0.254	A	AT	Zm00001d019546
**7**	47391027	0.58	0.463	A	G	Zm00001d019636
**7**	48345191	0.59	0.472	A	G	Zm00001d019656
**7**	48345190	0.54	0.346	G	A	Zm00001d019656
**7**	61638318	0.51	0.256	T	C	Zm00001d019789
**7**	64691322	0.57	0.42	T	C	Zm00001d019817
**7**	80236911	0.46	0.182	CA	C	Zm00001d019958
**7**	85222689	0.65	0.735	G	T	Zm00001d020008
**7**	85991042	0.53	0.32	G	A	Zm00001d020018
**7**	88881262	0.56	0.384	A	G	Zm00001d020053
**7**	102767126	0.48	0.215	C	T	Zm00001d020261
**7**	102767113	0.47	0.197	T	C	Zm00001d020261
**7**	109926506	0.59	0.475	A	C	Zm00001d020371
**7**	139313195	0.47	0.193	T	C	Zm00001d020985
**10**	97256557	0.47	0.19	A	G	Zm00001d024967
**10**	149763355	0.5	0.25	G	T	Zm00001d026678

**1**	203124624	0.47	0.191	T	C	Zm00001d031832
**1**	208903003	0.56	0.393	G	A	Zm00001d031989
**1**	208903001	0.47	0.196	G	A	Zm00001d031989
**1**	208903172	0.47	0.193	C	G	Zm00001d031989
**1**	208903175	0.47	0.193	T	C	Zm00001d031989
**1**	235327816	0.52	0.284	A	G	Zm00001d032704
**1**	300434957	0.51	0.27	G	A	Zm00001d034711
**6**	3352745	0.5	0.244	T	C	Zm00001d035056
**6**	36081296	0.5	0.25	CCTCCCTCCCCCT	C	Zm00001d035601
**6**	94241959	0.55	0.344	T	C	Zm00001d036610
**6**	114889551	0.58	0.452	G	C	Zm00001d037181
**6**	133276756	0.71	1.041	G	A	Zm00001d037659
**6**	133276741	0.69	0.919	A	AAT	Zm00001d037659
**6**	133276730	0.58	0.463	AT	A	Zm00001d037659
**6**	133276729	0.55	0.353	C	T	Zm00001d037659
**6**	145638815	0.47	0.19	C	T	Zm00001d038031
**6**	154622406	0.52	0.295	G	A	Zm00001d038328
**3**	19172923	0.49	0.224	C	T	Zm00001d039911
**3**	19384863	0.5	0.237	G	T	Zm00001d039920
**3**	20815188	0.46	0.183	T	TCAGTACCAAC	Zm00001d039963
**3**	34376474	0.59	0.475	T	G	Zm00001d040259
**3**	34376487	0.54	0.352	C	T	Zm00001d040259
**3**	34376503	0.51	0.276	C	T	Zm00001d040259
**3**	34376490	0.47	0.205	A	G	Zm00001d040259
**3**	87906262	0.52	0.276	C	T	Zm00001d040980
**3**	97014200	0.56	0.391	G	A	Zm00001d041092

**3**	106078597	0.49	0.239	T	C	Zm00001d041220
**3**	114814613	0.5	0.243	C	T	Zm00001d041379
**3**	121468298	0.48	0.208	G	A	Zm00001d041458
**3**	139073012	0.49	0.231	G	A	Zm00001d041816
**3**	139073038	0.47	0.191	T	C	Zm00001d041816
**3**	140445094	0.54	0.33	T	G	Zm00001d041846
**3**	140445069	0.53	0.316	C	T	Zm00001d041846
**3**	140445073	0.52	0.292	C	A	Zm00001d041846
**3**	140445100	0.5	0.25	C	A	Zm00001d041846
**3**	149693976	0.5	0.251	G	C	Zm00001d042074
**3**	156091089	0.53	0.325	A	G	Zm00001d042207
**3**	157883873	0.56	0.382	T	C	Zm00001d042262
**3**	159153262	0.5	0.25	G	T	Zm00001d042289
**3**	202092889	0.48	0.213	T	C	Zm00001d043502
**3**	226337960	0.62	0.573	T	A	Zm00001d044373
**3**	229746803	0.5	0.241	A	G	Zm00001d044498
**9**	20782777	0.46	0.182	C	T	Zm00001d045404
**9**	129735025	0.46	0.191	A	G	Zm00001d047428
**4**	156504659	0.47	0.198	G	C	Zm00001d051382
**4**	178261225	0.62	0.611	G	T	Zm00001d052064
**4**	178261213	0.58	0.463	G	C	Zm00001d052064
**4**	191344924	0.54	0.344	C	CT	Zm00001d052493
**4**	201128381	0.47	0.198	A	G	Zm00001d052792
**4**	225879205	0.47	0.19	C	T	Zm00001d053318
**4**	233512329	0.5	0.25	T	A	Zm00001d053542
**4**	243690332	0.55	0.361	C	G	Zm00001d053925

**4**	244608118	0.61	0.532	G	A	Zm00001d053986
**4**	244608103	0.58	0.477	G	A	Zm00001d053986
**4**	244612327	0.49	0.229	T	A	Zm00001d053987
**4**	244812965	0.62	0.625	G	T	Zm00001d053995

REF: The B73, ALT: Allelic variation.

Table S2. The list of primer for qPCR.

Name	Sequence
**ZmGAPDH-F**	CCCTTCATCACCACGGACTAC
**ZmGAPDH-R**	AACCTTCTTGGCACCACCCT
**Zm181-RT-F**	CAAAGCCAGCGCCACAAAG
**Zm181-RT-R**	GCGGTGATTATGCCACGAGTA
**Zm925-RT-F**	CTCAACACCTGGTTCCGCAC
**Zm925-RT-R**	CAGACGAAGAGCAGGACGTT
